# Mitochondrial Autoantibodies and the Role of Apoptosis in Pemphigus Vulgaris

**DOI:** 10.3390/antib11030055

**Published:** 2022-08-25

**Authors:** Dana M. Hutchison, Anna-Marie Hosking, Ellen M. Hong, Sergei A. Grando

**Affiliations:** 1 Department of Dermatology, University of California Irvine, Irvine, CA 92697, USA; 2 Beckman Laser Institute, University of California Irvine, Irvine, CA 92612, USA; 3 Department of Internal Medicine, Riverside Community Hospital, Riverside, CA 92501, USA; 4 Hackensack Meridian School of Medicine, Nutley, NJ 07110, USA; 5 Department of Biochemistry, University of California Irvine, Irvine, CA 92697, USA; 6 Institute for Immunology, University of California Irvine, Irvine, CA 92697, USA

**Keywords:** pemphigus vulgaris, apoptosis, apoptosis, antimitochondrial autoantibodies

## Abstract

Pemphigus vulgaris (PV) is an IgG autoantibody-mediated, potentially fatal mucocutaneous disease manifested by progressive non-healing erosions and blisters. Beyond acting to inhibit adhesion molecules, PVIgGs elicit a unique process of programmed cell death and detachment of epidermal keratinocytes termed apoptolysis. Mitochondrial damage by antimitochondrial antibodies (AMA) has proven to be a critical link in this process. AMA act synergistically with other autoantibodies in the pathogenesis of PV. Importantly, absorption of AMA inhibits the ability of PVIgGs to induce blisters. Pharmacologic agents that protect mitochondrial function offer a new targeted approach to treating this severe immunoblistering disease.

## 1. Introduction

Pemphigus encompasses a family of rare, potentially lethal autoimmune blistering dermatoses involving the skin and mucosal surfaces. The word ‘pemphigus’ derives from the Greek “pemphix”, which means blister. Its earliest use dates back to Hippocrates in 460–370 B.C. [1]. In modern history, the disease was first described by an Irish physician in 1788 [2], however, our current understanding of pemphigus pathophysiology began in 1964 with the discovery of autoantibodies in the sera of pemphigus vulgaris (PV) patients directed against the cell surface of keratinocytes [3]. The disease is associated with both circulating and tissue-bound IgG autoantibodies, and manifested by the loss of cell–cell adhesion of keratinocytes (acantholysis), and formation of non-healing suprabasal intraepidermal blisters.

IgG antibodies against desmoglein-1 (Dsg1) and desmoglein-3 (Dsg3), calcium-dependent cell adhesion molecules of the cadherin family, have been considered to play a primary role in the development of PV. However, explanation of the pathogenesis remains controversial [4]. Clinically, the detection of anti-Dsg3 reactivity, with or without anti-Dsg1 reactivity, is helpful in diagnosing PV. However, there have been a number of reports of patients in whom no reactivity was found, challenging the notion of an exclusive role these proteins have in the biologic mechanism of keratinocyte cohesion and their autoantibodies in blister formation in patients (reviewed in Ref. [5]).

Proteomic studies have led to the discovery of additional major defined types of non-Dsg proteins targeted by pemphigus autoantibodies, including: mitochondrial proteins, desmocollin 1 and 3 (Dsc1 and Dsc3), various nicotinic and muscarinic acetylcholine receptor subtypes, thyroid peroxidase, human leukocyte antigen (HLA) molecules, and secretory pathway Ca^2+^/Mn^2+^-ATPase isoform 1 (SPCA1) encoded by the ATP2C1 gene, which is mutated in Hailey-Hailey disease [6]. A “multiple hit” hypothesis has been proposed [7], wherein various non-Dsg autoantibodies against keratinocytes act synergistically with anti-Dsg autoantibodies to cause blistering. These non-Dsg autoantibodies can induce changes seen in PV, including keratinocyte shrinkage, cell–cell detachment, and triggering of apoptotic signaling events (reviewed in Ref. [4]). For example, non-Dsg autoantibodies against Dsc3, M3 muscarinic acetylcholine receptor (M3AR), and SPCA1 isolated from the sera of patients with anti-Dsg1/3 autoantibody-negative PV were found to be pathogenic, working synergistically with each other to cause acantholysis [8]. Thus, recent discoveries of numerous non-Dsg autoantibody species further develop our understanding of PV and implicate additional cell metabolism and signaling pathways involved in acantholysis [6,9].

In this article, we focus on targets of PV autoimmunity within the mitochondrion, as antimitochondrial antibodies (AMA) have proven to be a critical link in the pathogenesis of PV.

## 2. Apoptolysis

Beyond acting at the keratinocyte cell membrane to block the function of adhesion molecules, PVIgGs elicit the signaling events that trigger the keratinocyte cell death program. The term “apoptolysis” has been coined to describe the distinct autoantibody-induced process of keratinocyte structural damage and detachment (acantholysis) followed by death (apoptosis), which is unique to PV. Acantholysis and apoptosis are inseparable in PV and are mediated by the same cell death enzymes [10]. Apoptosis refers to programmed cell death—a pathway not activated by inflammation, but rather by cysteine aspartate proteases, or caspases. This cell death pathway can be activated by cellular damage (intrinsic pathway) or by signaling molecules (extrinsic, death receptor-initiated pathway), and ultimately results in the formation of apoptotic bodies which are then cleared by phagocytic cells [11]. The best-known cell death pathways are apoptosis, oncosis, and necrosis, however others have recently been described by the Nomenclature Committee on Cell Death (NCCD) [12]. Additional, newly described apoptolytic pathways that play a role in the pathophysiology of skin blistering characteristic of PV should be further investigated.

Mitochondria play a critical role in programmed cell death (reviewed in Ref. [13]). Initiation of apoptotic pathways ultimately disrupt the inner mitochondrial membrane, resulting in loss of the mitochondrial transmembrane potential and leakage of pro-apoptotic proteins into the cell cytosol, including the release of cytochrome c (CytC), a marker for mitochondrial outer membrane permealization and early apoptosis, and subsequent activation of caspases [14]. The disruption of mitochondrial energy production, combined with cleavage of adhesion and structural molecules, causes cytoskeleton collapse and the keratinocyte to shrink [10]. The fundamental feature of apoptolysis is that anti-keratinocyte antibodies in PV cause basal keratinocytes only to shrink, but not to die, giving rise to their “tombstone” appearance on histopathology. This is distinct from the classic apoptotic processes in the epidermis of patients with Stevens-Johnson Syndrome/Toxic Epidermal Necrolysis, in which apoptosis leads to sloughing of the entire epidermis, including its basal layer [15].

In PV, the apoptotic pathway is activated long before morphological evidence of acantholysis [16,17]. The hypothetical sequence of apoptolysis development in PV has five consecutive steps: (i) Pathogenic autoantibodies bind PV antigens on the surface of keratinocytes and pro-apoptolytic signals are transduced. (ii) Activation of EGFR, mTOR, Src, p38 MAPK and other signaling pathways increase intracellular calcium and initiate programmed cell death enzymatic cascades predominately in basal keratinocytes. (iii) Executioner caspases cleave tonofilaments, leading to their collapse and retraction, while inter-desmosomal adhesion complexes are phosphorylated and dissociate. This results in basal cell contraction, a crossing step of both the apoptotic and early acantholytic pathways. The majority of desmosomes remain intact and bridge collapsing keratinocytes. (iv) The continued degradation of structural proteins by the programmed cell death enzymes lead to cytoskeleton collapse and complete separation of shrinking keratinocytes (visible acantholysis). The sloughed cell membrane pieces trigger production of scavenging (secondary) autoantibodies to Dsg, Dsc, E-cadherin and other adhesion molecules attached to the cell membrane. (v) The end result is rounding up and apoptotic death of acantholytic cells resulting from irreversible damage to mitochondrial and nuclear proteins by the same cell death enzymes giving rise to a “tombstone” appearance of the surviving basal keratinocytes [10]. The more recent observations of the pathogenic role of AMA, however, indicate that damage of mitochondria occurs at an early stage of apoptolysis.

## 3. Mitochondrial Damage by AMA in PV

Patients with PV produce PVIgG antibodies targeting a variety of proteins, including those at the inner and outer mitochondrial membrane, as well as the mitochondrial matrix [18,19,20]. Utilizing protein microarray, the most common antigen targets recognized by AMA in PV have been identified from a large cohort of patients (Table 1) [18]. Based on the known functions of these proteins, AMA likely lead to mitochondrial dysfunction by altering cellular ability to produce or inactivate reactive oxygen species, perform oxidative phosphorylation, and participate in oxygen respiration. The exact mechanism of mitochondrial damage likely varies greatly among PV patients, consistent with the strikingly wide spectrum of disease severity and treatment response [20]. Importantly, absorption of AMA inhibits the ability of PVIgGs to induce keratinocyte detachment and blistering [19].

**Figure 1:**
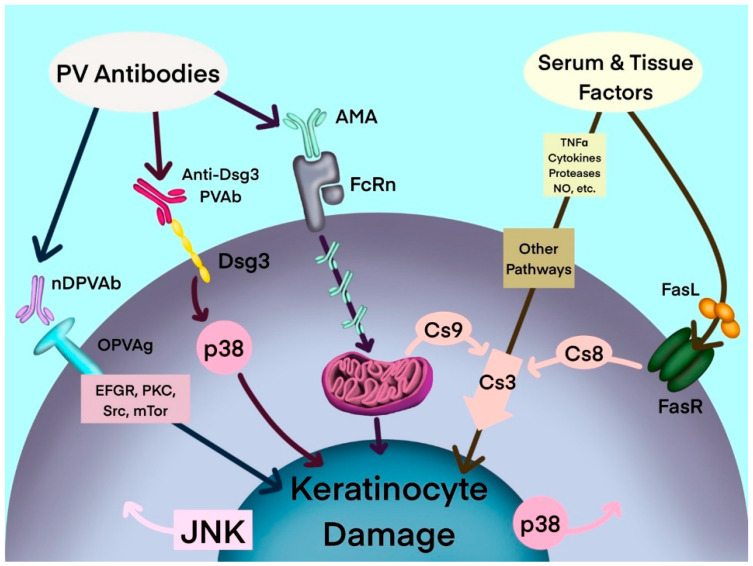
Hypothetical multipathogenic mechanism of interconnected signaling cascades leading to keratinocyte apoptolysis in pemphigus (modified from Marchenko et al. [19]). AMA: antimitochondrial antibodies; Anti-Dsg3 PVAb: anti-desmoglein 3 PV antibody; Cs: caspase; Dsg3: desmoglein-3; EGFR: epidermal growth factor receptor; FasL: Fas ligand; FasR: Fas receptor; JNK: c-Jun N-terminal kinase; mTOR: mammalian target of rapamycin; NO: nitric oxide; nDPVAb: non-Dsg PV antibodies; OPVAg: other PV antigens; PKC: protein kinase C; PVAb: PV antibody; Src: SRC proto-oncogene, nonreceptor tyrosine kinase; TNF-α: tumor necrosis factor-α.

Antibodies to mitochondrial proteins, among anti-keratinocyte antibodies and several other soluble pathogenic factors, act synergistically to activate keratinocyte cell death pathways in PV (Figure 1). In an organ culture of neonatal mouse skin—an in vitro model of PV—AMA and anti-Dsg1/3 autoantibodies acted synergistically to induce acantholysis [21]. In that study, treatment with AMA alone did not result in acantholysis, however the combination of AMA and a mixture of anti-Dsg antibodies induced acantholysis. The AMA/anti-Dsg3 combination induced epidermal splitting suprabasally and the AMA/anti-Dsg1 combination induced epidermal splitting subcorneally, which is characteristic of pemphigus foliaceus. Moreover, while acantholysis was observed following treatment with high concentrations of the human anti-Dsg single-chain variable fragment (scFv), at low, physiologic doses the scFv was not able to induce keratinocyte detachment. Acantholysis was only observed when it was combined with AMA [21]. The synergy of AMA with other autoantibodies in PV implies that a simultaneous hit is required to alter the keratinocyte ability to maintain epidermal integrity. It is theorized that the binding of a single type of autoantibody only induces reversible changes in the keratinocyte, such that the cell maintains its ability to recover via self-repair mechanisms. Irreversible keratinocyte damage only occurs following presumed synchronized inactivation of salvage pathways by partnering autoantibodies, leading to loss of epidermal integrity [21].

Human skin contains a complex non-neuronal cholinergic network composed of the cytotransmitter acetylcholine and its nicotinic and muscarinic receptors [22]. These receptors are involved in keratinocyte cell–cell and cell-matrix adhesion and some are targeted by PV autoantibodies (reviewed in Ref. [6]). In a neonatal mouse model of PV, preabsorption of PVIgGs with recombinant pemphaxin, a low-affinity dual muscarinic and nicotinic receptor for acetylcholine, eliminated the acantholytic activity of PVIgGs. In turn, the acantholytic activity could be restored by the addition of anti-pemphaxin antibody back to the preabsorbed PVIgG fraction [23]. In addition to being present on the surface of keratinocytes, nicotinic acetylcholine receptors have been found on the mitochondrial outer membrane (mt-nAChRs) and are one of the targets of AMA. Stimulation of mt-nAChR prevents apoptosis by inhibiting mitochondrial permeability transition pore (mPTP) opening, thus preventing CytC release from the organelle [20]. Interestingly, nicotinergic stimulation has been shown to protect keratinocytes from apoptolysis [20].

Among non-Dsg autoantibodies in PV patient sera, that increase activity of pathways involved in apoptolysis, a combination of anti-M3AR, anti-SPCA1 and Dsc3 has been identified [8]. When each of the above autoantibodies was tested alone in a neonatal mouse skin explant model, none were able to solely induce acantholysis. However, a mixture of all three produced an acantholytic effect similar to that of PVIgGs. When the combination was further tested in a model of PV in BABL/c mice, it was also found to be sufficient to disrupt epidermal integrity in vivo [8]. Thus, antibodies altering vital cell functions (i.e., anti-M3AR), cell adhesion (i.e., anti-Dsc3) and Ca^2+^ metabolism (i.e., anti-SPCA1) appear to work synergistically to produce an acantholytic effect similar to that of total PVIgGs in a clinically relevant manner.

It has been shown that the binding of anti-M3AR or anti-SPCA1 autoantibodies to keratinocytes leads to mitochondrial damage and the release of CytC and activation of the caspase 9 pathway [24]. Anti-SPCA1 produced a 10-fold, and anti-M3AR autoantibody a 4–5 fold, increase in levels of CytC and Cs-9, respectively [24]. Further, anti-M3AR and anti-SPCA1 autoantibodies worked synergistically in a 3D culture of human epidermis. A mixture of anti-SPCA1 and anti-M3AR autoantibodies resulted in changes in the morphology of human epidermis consistent with acantholysis, including “bubbling” at the epidermis while basal cells remained intact at the dermal-epidermal junction [24]. However, when given alone, anti-M3AR did not cause morphological changes in that in vitro model, while anti-SPCA1 given alone resulted in shrinkage of epidermal keratinocytes [24].

SPCA1 is located on the Golgi apparatus, and it is thought that a defect in SPCA1 Ca^2+^ sequestration contributes to Golgi stress leading to apoptosis [25]. The Golgi complex is capable of transducing pro-apoptotic signals which are partially mediated through caspase 2 (Cs-2) that localizes to the Golgi apparatus [26,27]. A recent study demonstrated that the effects of PV anti-SPCA1 autoantibody on mitochondrial CytC release were abolished in the presence of a Cs-2 inhibitor [24]. These findings suggest that anti-SPCA1 autoantibodies alter mitochondrial function through Cs-2, triggering early pro-apoptotic events. Notably, SPCA1 is encoded by the *ATP2C1* gene, which is mutated in benign chronic pemphigus (also known as Hailey-Hailey disease) [28].

The neonatal Fc receptor (FcRn) may, in part, mediate the pathogenic effects of PV autoantibodies to intracellular self-antigens, including SPCA1 and those present in mitochondria. Following binding of PVIgG to FcRn on the cell membrane of keratinocytes, complexes of PVIgG-FcRn are internalized and trafficked to the mitochondria, where they are released from endosomes [21]. The complexes dissociate and AMA reach mitochondria, triggering early apoptotic events and cell shrinkage. However, this AMA-induced damage is reversible. Interestingly, cells lacking FcRn do not internalize PVIgGs and AMA is therefore unable to reach the mitochondria [21]. Further, pretreatment of mouse keratinocytes with anti-FcRn antibody, which functionally inactivates FcRn, prevents shrinkage of keratinocytes as well as other AMA-dependent changes in mitochondrial integrity and metabolism [21]. Since FcRn is predominantly expressed in the basal epidermal layer [29], basal and suprabasal keratinocytes should respond differently to the PVIgGs entering keratinocytes via FcRn-mediated mechanism. This may explain the suprabasal location of epidermal split in PV, as only basal keratinocytes shrink, thereby separating themselves from suprabasal keratinocytes. However, the exact mechanism by which PVIgGs enter keratinocytes to reach the mitochondrial target antigens, and why other cell types in the body that contain the same mitochondrial antigens are not affected by PVIgGs, is still unknown.

New research has implicated that the thioredoxin-2 (Trx2)/apoptosis signal-regulating kinase 1 (ASK1) pathway may play a key role in mediating mitochondrial injury in PV [30]. ASK1, a serine/threonine kinase, is activated by oxidative stress and triggers apoptosis. One of the functions of Trx2 is to inactivate ASK1 by forming a complex with the molecule, thereby preventing its phosphorylation and activation. Elevated levels of reactive oxygen species, which are found following mitochondrial injury by AMA, oxidize the cysteine residues of Trx2, promoting the dissociation of the Trx2-ASK1 complex and allowing activated ASK1 to trigger apoptotic events. In an in vitro study of keratinocytes cultured with PV sera, the Trx2/ASK1 cascade was abnormally activated, with decreased local expression of Trx2, an increased amount of phosphorylated ASK1, and an increased rate of apoptosis compared to control cells [29]. In a mouse model, the overexpression of Trx2 decreased ASK1 phosphorylation, the apoptotic rate, and relieved acantholysis and blister formation. Thus, Trx2 appears to have a protective role in mitochondrial injury and compounds targeting the Trx2/ASK1 pathway may help prevent progression of PV in the future.

## 4. Efficacy of Mitochondrion Protective Agents in Pemphigus Patients

While the antigen specificities of AMA produced by individual PV patients is highly variable (Table 1), uniform protection from mitochondrial damage can be achieved with nonsteroidal mitochondrion-protective pharmacologic agents [18]. Growing evidence suggests that the use of mitochondrion-protecting drugs, such as cyclosporine A (CsA), tetracyclines and nicotinamide (also called niacinamide), are justified in the treatment of PV [18]. In addition to inhibiting the production of cytokines involved in T-cell activation, CsA can protect mitochondria by binding cyclophilin D and inhibiting opening of mPTP, allowing the mitochondrion to retain a high transmembrane potential (Δψm) [31,32,33]. Tetracyclines, such as minocycline and doxycyline, inhibit mPTP opening by reducing mitochondrial uptake of Ca^2+^, inhibiting loss of Δψm and preventing CytC release [34]. Niacinamide, one of the two principle forms of vitamin B_3_, is a precursor of the coenzyme NAD^+^ consumed during ATP generation in the mitochondrial electron transport chain. Vitamin B_3_ is thought to help cells retain high-quality mitochondria by activating autophagy of mitochondria with low Δψm, which indicates a damaged (depolarized) cell [33]. Animal studies provided evidence that pharmacologic protection of mitochondria with CsA, minocycline, and nicotinamide prevents PVIgGs-mediated induction of skin blisters in mouse skin [18], which is in keeping with clinical reports that PV lesions can be partially controlled by these agents in the absence of systemic steroids [35,36,37].

One interesting observation is that nicotine competes with PVIgGs for binding to mt-nAChRs, thereby inhibiting mitochondrial CytC release in a dose-dependent fashion, and prevents intrinsic apoptosis in keratinocytes [20]. The potential therapeutic effect of nicotinergic stimulation in PV has been reported in a case study [38] as well as in epidemiological data showing a beneficial effect of smoking on PV [39,40,41]. This may be in part due to nicotinic agonism at mt-nAChRs protecting mitochondria.

Additionally, sirolimus (also known as rapamycin) has been proposed to protect keratinocytes from PVIgG aggression through a poorly understood mechanism. In an experimental setting, pretreatment with sirolimus prevented acantholysis in a mouse model of PV [42]. In a clinical setting, within two weeks of initiating therapy with sirolimus, PV lesions on a man with severe side effects to prednisone completely healed, allowing him to rapidly taper off prednisone and remain lesion free on a maintenance dose of 2 mg/day of sirolimus. Studies in other mitochondrial disorders have suggested that sirolimus improves cellular function by reducing the number of dysfunctional mitochondria within an organelle, thereby preserving mitochondrial integrity [43].

At the UC Irvine Immunobullous Clinic, PV patients are successfully treated with a multidrug therapeutic approach including mitochondrion-protecting agents (minocycline or doxycycline 200 mg/day + niacinamide 1.5 g/day), in addition to intravenous immunoglobulin, or IVIg, systemic corticosteroids, and an immunosuppressive cytotoxic drug (mycophenolate mofetil, azathioprine or cyclophosphamide) [44]. The synergy of the drugs utilized in this protocol allows for rapid achievement and maintenance of clinical remission in approximately 88% of pemphigus patients with a smaller than usual cumulative dose of systemic corticosteroids. Indeed, while these mitochondrion protective agents are already utilized in the treatment of PV, novel pharmacologic prospects which protect and or compensate for disrupted mitochondrial function may offer an even safer, nonsteroidal approach to treating PV in the future.

## 5. Conclusions and Future Directions

The acantholytic process in PV is complex and involves autoantibodies directed against various keratinocyte proteins that maintain adhesion and other vital cell functions. While different pathogenic autoantibodies act synergistically in the pathogenesis of PV, pharmacological protection or the elimination of a single antibody may suffice to protect epidermal integrity and halt development of the disease. Further characterization of the role of individual AMA causing mitochondrial injury in the pathogenesis of PV may lead to development of personalized pharmacologic therapies to correct mitochondrial abnormalities unique to individual PV patients. Future studies to improve our understanding of the immunopathogenesis of PV should, therefore, aid in the development of novel and more efficient therapeutic modalities.

## Figures and Tables

**Table 1:** Mitochondrial autoantibodies in patients with PV (adapted from Kalantari et al. [18]) *.

Symbol	Antigen	Localization on Mitochondria	Frequency	
			PV (%)	Control (%)
ABAT-V1	4-Aminobutyrate aminotransferase, mitochondrial; 50 kDa	Matrix	19	4
ALDH4A1	Aldehyde dehydrogenase 4 family, member A1	Matrix	23	5
CPT1B	Carnitine *O*-palmitoyltransferase 1B	Outer membrane	18	5
CRAT	Carnitine *O*-acetyltransferase	Inner membrane	28	7
CYB5B	Cytochrome *b*_5_ type B; 21 kDa	Outer membrane	19	1
ETFA	Electron transfer flavoprotein, α protein	Matrix	19	4
ETFB	Electron transfer flavoprotein, β protein	Matrix	21	3
FDXR-V2	NADPH:adrenodoxin oxidoreductase	Matrix	25	6
FH	Fumarate hydratase (fumarase)	Mitochondrion	29	3
MAOB	Amine oxidase (flavin-containing) B	Outer membrane	27	5
ME2	NAD-dependent malic enzyme	Matrix	18	6
ME3	NADP-dependent malic enzyme, mitochondrial	Matrix	23	8
MLYCD	Malonyl-CoA decarboxylase	Mitochondrion	29	4
NDUFA9	NADH dehydrogenase [ubiquinone] 1α subcomplex subunit 9; 39 kDa	Matrix	20	3
NDUFA13	NADH dehydrogenase [ubiquinone] 1α subcomplex subunit 13; 16 kDa	Inner membrane	24	6
NDUFB10	NADH dehydrogenase [ubiquinone] 1β subcomplex subunit 10	Matrix	17	2
NDUFV3	NADH dehydrogenase [ubiquinone] flavoprotein 3; 9 kDa	Inner membrane	19	4
NDUFS6	NADH dehydrogenase [ubiquinone] iron-sulfur protein 6; 13 kDa	Inner membrane	24	6
PC	Pyruvate carboxylase	Matrix	32	5
PDK4	Pyruvate dehydrogenase kinase, isozyme 4	Matrix	24	4
PDHA1	Pyruvate dehydrogenase E1 component α subunit, somatic form	Glycolysis	30	3
PMPCB	Mitochondrial processing peptidase β subunit	Mitochondrial organization	31	4
PRODH	Proline oxidase	Matrix	25	6
SOD2	Superoxide dismutase [Mn]	Matrix	23	2
TIMM44	Mitochondrial import inner membrane translocase subunit	Inner membrane	20	4

* Every PV serum analyzed in the referenced study contained an autoantibody to at least one mitochondrial protein (data not shown).

## Data Availability

No new data were created or analyzed in this study. Data sharing is not applicable to this article.
